# Mobile Colistin Resistance Enzyme MCR‐3 Facilitates Bacterial Evasion of Host Phagocytosis

**DOI:** 10.1002/advs.202101336

**Published:** 2021-07-29

**Authors:** Wenjuan Yin, Zhuoren Ling, Yanjun Dong, Lu Qiao, Yingbo Shen, Zhihai Liu, Yifan Wu, Wan Li, Rong Zhang, Timothy R. Walsh, Chongshan Dai, Juan Li, Hui Yang, Dejun Liu, Yang Wang, George Fu Gao, Jianzhong Shen

**Affiliations:** ^1^ Beijing Key Laboratory of Detection Technology for Animal‐Derived Food Safety College of Veterinary Medicine China Agricultural University Beijing 100193 China; ^2^ College of Basic Medical Science Key Laboratory of Pathogenesis Mechanism and Control of Inflammatory‐Autoimmune Diseases of Hebei Province Hebei University Baoding 071002 China; ^3^ Department of Basic Veterinary Medicine College of Veterinary Medicine China Agricultural University Haidian Beijing 100193 China; ^4^ CAS Key Laboratory of Pathogenic Microbiology and Immunology Institute of Microbiology Chinese Academy of Sciences (CAS) Beijing 100101 China; ^5^ Agricultural Bio‐Pharmaceutical Laboratory College of Chemistry and Pharmaceutical Sciences Qingdao Agricultural University Qingdao 266109 China; ^6^ The Second Affiliated Hospital of Zhejiang University Zhejiang University Hangzhou 310009 China; ^7^ Department of Zoology University of Oxford Oxford OX1 3SZ UK; ^8^ State Key Laboratory of Infectious Disease Prevention and Control National Institute for Communicable Disease Control and Prevention Chinese Center for Disease Control and Prevention Changping Beijing 102206 China; ^9^ NHC Key Laboratory of Food Safety Risk Assessment China National Center for Food Safety Risk Assessment No. 7 Panjiayuan Nanli Beijing 100021 China; ^10^ College of Veterinary Medicine China Agricultural University Haidian Beijing 100193 China

**Keywords:** colistin, lipid A modification, *mcr‐3*, phagocytosis, virulence

## Abstract

Mobile colistin resistance enzyme MCR‐3 is a phosphoethanolamine transferase modifying lipid A in Gram‐negative bacteria. MCR‐3 generally mediates low‐level (≤8 mg L^−1^) colistin resistance among Enterobacteriaceae, but occasionally confers high‐level (>128 mg L^−1^) resistance in aeromonads. Herein, it is determined that MCR‐3, together with another lipid A modification mediated by the *arnBCADTEF* operon, may be responsible for high‐level colistin resistance in aeromonads. Lipid A is the critical site of pathogens for Toll‐like receptor 4 recognizing. However, it is unknown whether or how MCR‐3‐mediated lipid A modification affects the host immune response. Compared with the wild‐type strains, increased mortality is observed in mice intraperitoneally‐infected with *mcr‐3*‐positive *Aeromonas salmonicida* and *Escherichia coli* strains, along with sepsis symptoms. Further, *mcr‐3*‐positive strains show decreased clearance rates than wild‐type strains, leading to bacterial accumulation in organs. The increased mortality is tightly associated with the increased tissue hypoxia, injury, and post‐inflammation. MCR‐3 expression also impairs phagocytosis efficiency both in vivo and in vitro, contributing to the increased persistence of *mcr‐3*‐positive bacteria in tissues compared with parental strains. This study, for the first time, reveals a dual function of MCR‐3 in bacterial resistance and pathogenicity, which calls for caution in treating the infections caused by *mcr*‐positive pathogens.

## Graphical abstract

The synergistic lipid A modifications by mobile colistin resistance gene (*mcr‐3*) and *arnBCADTEF* operon are responsible for high‐level colistin resistance in *Aeromonas salmonicida*. Both *Escherichia coli* and *A. salmonicida* strains harboring *mcr‐3* suffer less phagocytosis and present enhanced pathogenicity. The dual threat, increased colistin resistance and pathogenicity, of infections caused by MCR‐producing pathogens needs considerable attention in the clinical settings.

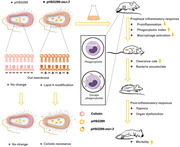

## 1 Introduction

Colistin is considered as the “last resort” antibiotic for the treatment of infections caused by extensively drug‐resistant Gram‐negative bacteria.^[^ ^1^, ^2^ ^]^ However, since 2015, the efficiency of colistin has been largely compromised by the emergence and rapid dissemination of mobile colistin resistance (*mcr*) genes among Enterobacteriaceae and *Aeromonas* species.^[^ ^3^, ^4^ ^]^ Of the ten plasmid‐mediated *mcr* gene variants characterized to date (*mcr‐1* to *mcr‐10*), *mcr‐1*, *mcr‐3*, and *mcr‐9* are the most frequently and globally identified.^[^ ^4^, ^5^, ^6^ ^]^ *mcr‐3* largely exists in aquatic animal‐borne *Aeromonas* species, which are regarded as a reservoir for other *mcr* variants.^[^ ^7^, ^8^ ^]^

*mcr* genes encode phosphoethanolamine (pEtN) transferases that modify the lipid A moieties of lipopolysaccharide (LPS) in the outer membranes of Gram‐negative bacteria, which reduces the net negative charge of LPS, and thereby lowering its affinity for colistin.^[^ ^9^, ^10^, ^11^ ^]^ The transmembrane region, catalytic domain, and inside linker between them were determined by the function of MCR‐3 on lipid A modification.^[^ ^12^ ^]^ This modification of lipid A normally mediates low‐level colistin resistance (minimum inhibitory concentrations (MICs) ≤8 mg L^−1^) in Enterobacteriaceae;^[^ ^3^, ^13^, ^14^ ^]^ however, some MCR‐producing *Aeromonas* spp. demonstrate high levels of resistance (MIC values ranging from 32 to >128 mg L^−1^).^[^ ^4^, ^11^, ^15^ ^]^ Acquisition of a single *mcr‐3* gene by *Aeromonas salmonicida* increased the MIC of colistin from 1 to 64 mg L^−1^.^[^ ^16^ ^]^ However, the mechanism underlying this high‐level colistin resistance in *mcr*‐carrying *Aeromonas* spp. remains unknown.

Phagocytes, including monocytes, macrophages, and neutrophils, are the major bacterial eliminators of the host innate immune system.^[^ ^17^ ^]^ Toll‐like receptor 4 (TLR4), a pattern recognition receptor, has the critical function of recognizing pathogens and triggering immune responses via the transduction of downstream signals such as nuclear factor kappa‐light‐chain‐enhancer of activated B cells (NF‐*κ*B).^[^ ^17^ ^]^ Bacterial LPS is one such structure that is recognized by recognition receptors and activates phagocytosis.^[^ ^18^ ^]^ Wild‐type LPS stimulates a much stronger immune response than modified LPS,^[^ ^19^, ^20^ ^]^ which has been shown to be a crucial factor in impaired LPS recognition. Therefore, in addition to altering the bacterial resistance profile, modification of lipid A also plays a vital role in protecting bacteria from host immune defenses through affecting recognition by the TLR4‐myeloid differentiation‐2 (TLR4‐MD2) receptor.^[^ ^20^ ^]^ For instance, inactivation of the lipid A synthesis pathway in *Acinetobacter baumannii*,^[^ ^21^ ^]^ hydroxylation of the lipid A acyl chain in *Klebsiella pneumoniae*,^[^ ^22^ ^]^ and addition of an aminoarabinose to the phosphate group of lipid A in *Salmonella enterica* serovar Typhimurium have all led to weakened host inflammatory responses and decreased bacterial clearance.^[^ ^23^ ^]^ In addition, modification of lipid A changes the overall charge of the bacterial cell surface and thus promotes resistance to cationic antimicrobial peptides, which are important components of the host innate immune response.^[^ ^20^ ^]^ Therefore, these modifications protect bacteria by enabling them to elude the host immune surveillance.^[^ ^20^, ^24^, ^25^, ^26^ ^]^

Previous study revealed that MCR‐1‐modified *Escherichia coli* LPS induced weaker macrophage response than native LPS.^[^ ^26^ ^]^ However, whether MCR‐mediated lipid A modification provides an altered immune escape mechanism in vivo and thus increases the severity of infection in healthy hosts is not well understood. Herein, we explore the molecular mechanisms responsible for the high‐level colistin resistance displayed by *mcr‐3*‐positive *Aeromonas* strains. By using murine infection models, we investigate the changes in the virulence of *Aeromonas* species and Enterobacteriaceae resulting from *mcr‐3*‐mediated modification in lipid A. Our data provides evidence that *mcr‐3*‐mediated lipid A decoration leads to enhanced bacterial pathogenicity by enhancing their ability to escape from innate immune defenses.

## 2 Results

### 2.1 *mcr‐3* Confers Higher Levels of Colistin Resistance in *A. salmonicida* than in *E. coli*

**Figure 1:**
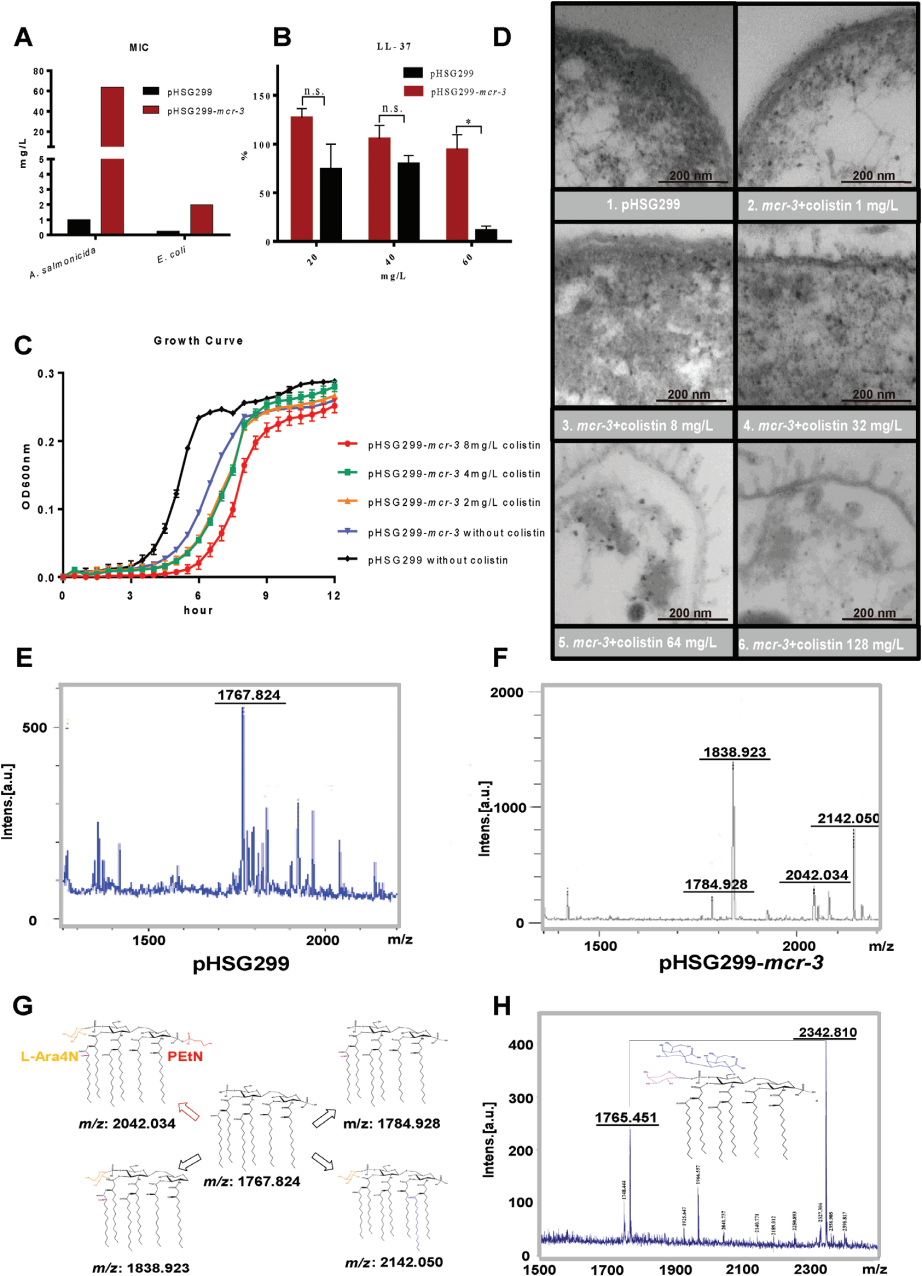
Phenotype and colistin resistance mechanism of the *mcr‐3*‐positive AS1 strain. A) MICs of colistin against *A. salmonicida* AS1 and *E. coli* DH5*α* harboring the recombinant *mcr‐3*‐carrying plasmid or empty vector. B) Comparison of the survival rates (%) of strain AS1 with/without *mcr‐3* in the presence of different concentrations of LL‐37 (two‐tailed unpaired *t*‐test; ^*^ *P* < 0.05). C) Growth curve of strain AS1 with/without *mcr‐3*. The *mcr‐3*‐positive strain was cultivated in the presence of colistin at concentrations of 0, 2, 4, or 8 mg L^−1^. D) TEM micrographs of AS1 with/without *mcr‐3*. The *mcr‐3*‐positive strains were treated with colistin at concentrations of 1, 8, 32 (1/2‐fold of MIC), 64 (1‐fold of MIC), or 128 mg L^−1^ (2‐fold of MIC). E) Negative‐ion MALDI‐TOF mass spectrometry analysis of strain AS1 carrying plasmid pHSG299. F) Negative‐ion MALDI‐TOF mass spectrometry analysis of strain AS1 carrying plasmid *mcr‐3*‐pHSG299. G) Chemical structure analysis of modified lipid A from strain AS1. H) Mass spectrometry analysis of *Aeromonas caviae* harboring plasmid pT. The structure shows the *arnT*‐mediated lipid A modification.

*A. salmonicida* strain AS1, used in this study as a model bacterium, was subjected to antimicrobial susceptibility testing and whole‐genome sequencing. The antibiotic resistance gene profile of strain AS1 correlated well with the results of MIC testing using 12 antimicrobial agents (Tables S1 and S2, Supporting Information). Our results indicated that AS1 harboring plasmid pHSG299‐*mcr‐3* exhibited high‐level colistin resistance (64‐fold increase in resistance compared with the wild‐type strain) (**Figure** **1A**), while the MIC of colistin against an *E. coli* strain harboring the same plasmid was only 8‐fold higher than that against the wild‐type strain.^[^ ^14^, ^16^ ^]^ Interestingly, increased transcription of *mcr‐3* was observed in *E. coli* DH5*α* compared with *A. salmonicida* AS1 (Figure S1B, Supporting Information), suggesting an additional colistin resistance mechanism may exist in strain AS1, contributing to the high‐level resistance. In addition, based on analysis of half maximal inhibitory concentrations, *mcr‐3*‐positive AS1 was also resistant to human antimicrobial peptide LL‐37 (Figure 1B).

Acquisition of *mcr‐3* changed the membrane potential of strain AS1 as a result of lipid A modification. Additionally, the physiological function of the cell is also likely to be altered. AS1 harboring empty vector pHSG299 reached the exponential growth phase at 3 h postinoculation, while AS1 harboring pHSG299‐*mcr‐3* reached the same growth phase at 5 h postinoculation, indicating that MCR‐3 expression retards the growth rate of strain AS1. In addition, the growth rate of *mcr‐3*‐positive AS1 was not significantly altered by the addition of colistin at any of the tested concentrations (Figure 1C). Transmission electron microscopy (TEM) was subsequently used to examine the cellular morphology of strain AS1 harboring *mcr‐3* grown under increasing concentrations of colistin. At lower concentrations, the *mcr‐3*‐positive strain showed a similar morphology to the wild‐type strain (Figure 1D). However, the addition of colistin at concentrations equivalent to 0.5× (32 mg L^−1^), 1× (64 mg L^−1^), and 2× (128 mg L^−1^) the MIC resulted in altered cellular morphology. Cells treated with colistin at concentrations equivalent to or greater than the MIC produced pilus‐like structures around the bacterial membrane, which may play a role in cellular defense when stimulated with colistin.

### 2.2 High Colistin MICs Can Be Attributed to Synergistic Lipid A Modifications Mediated by *mcr‐3* and *arnBCADTEF*

To investigate the mechanism responsible for the high‐level colistin resistance phenotype, lipid A from strain AS1 with/without the *mcr‐3*‐mediated modification were extracted and analyzed by matrix‐assisted laser desorption/ionization time‐of‐flight (MALDI‐TOF) mass spectrometry. The initial ion of lipid A from *A. salmonicida* was observed as a peak formed at *m*/*z* 1767.824 (Figure 1E).^[^ ^27^ ^]^ Acquisition of recombinant plasmid pHSG299‐*mcr‐3* resulted in four additional peaks, observed at *m*/*z* 1784.928, *m*/*z* 1838.923, *m*/*z* 2042.034, and *m*/*z* 2142.050, which were a consequence of lipid A modification (Figure 1F). The peak observed at *m*/*z* 2042.034 corresponded to the initial ion (*m*/*z* 1767.824) with the addition of 4‐amino‐4‐deoxy‐l‐arabinose (l‐Ara4N, *m*/*z* 132) and pEtN (*m*/*z* 123) groups at the 1‐ and 4‐phosphate groups of lipid A,^[^ ^28^ ^]^ respectively (Figure 1G).

We have confirmed that the MCR‐3 can mediate the addition of pEtN.^[^ ^29^ ^]^ While the l‐Ara4N modification is associated with the protein products of the *arnBCADTEF* operon in both Enterobacteriaceae and *Pseudomonas* species.^[^ ^30^ ^]^ Therefore, we cloned this operon into pHSG299 and transformed it into *Aeromonas caviae* strain ZJ66‐1 (Figure S1A, Supporting Information). Compared with strain ZJ66‐1P (harboring pHSG299), strain ZJ66‐1T (harboring pHSG299‐*arnBCADTEF*) showed a 32‐fold increase in colistin MIC (from 1 to 32 mg L^−1^). Lipid A extracted from ZJ66‐1T showed an additional peak at *m*/*z* 2342.810 (Figure 1H), suggesting a possible synergistic effects from the *arnBCADTEF* operon and *mcr‐3* in aeromonads. In addition to these modifications, the ion observed at *m*/*z* 2142.050 had palmitate (*m*/*z* 239) and hydroxyl (*m*/*z* 16) residues.^[^ ^18^ ^]^ The peak at *m*/*z* 1838.928 might result from the loss of a phosphate group during the lipid A extraction process, for which extensive boiling was required.^[^ ^31^ ^]^ These lipid A modifications were likely to contribute to the high‐level colistin resistance of *A. salmonicida* and may explain why the acquisition of *mcr‐3* by *A. salmonicida* results in increased colistin MICs (from 1 to 64 mg L^−1^) compared with those observed for Enterobacteriaceae.

### 2.3 *mcr‐3*‐Positive Bacteria Induce More Severe Tissue Injury and Higher Mortality Rates in Mice

**Figure 2:**
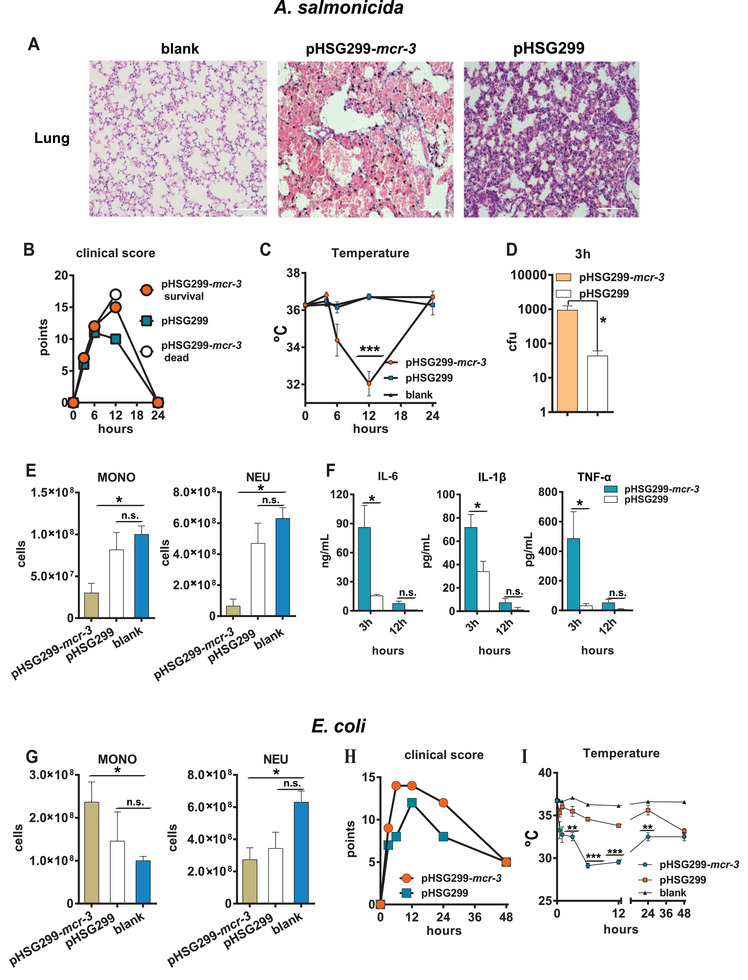
*mcr‐3*‐positive bacteria showed increased pathogenicity in mice compared with the *mcr‐3*‐negative strain. A) Pathological sections of lung removed from dead mice. B) Clinical scores of infected mice. The overall status of each mouse was assessed at each time point. C) Body temperatures of infected mice (three mice were tested at each time point; ^***^ *P* < 0.001). D) Bacterial counts per 100 µL of blood (three mice were tested at 3 h postinfection; ^*^ *P* < 0.05). E) Enumeration of monocytes and neutrophils per liter of blood (three mice were tested at 6 h postinfection; ^*^ *P* < 0.05). F) Levels of IL‐6, IL‐1*β*, and TNF‐*α* expression in serum at 3 and 12 h postinfection (three mice were tested at each time point; ^*^ *P* < 0.05). G) Enumeration of monocytes and neutrophils per liter of blood (three mice were tested at 6 h postinfection; ^*^ *P* < 0.05). H) Clinical scores of mice. The overall status of each mouse was assessed at each time point. I) Body temperatures of mice (three mice were tested at each time point; ^**^ *P* < 0.01 and ^***^ *P* < 0.001). Statistical significance was assessed by two‐tailed unpaired *t*‐test.

To determine the clinical importance of *mcr‐3*‐carrying AS1, BALB/c mice were challenged with 200 µL *mcr‐3*‐positive/negative strains at three different concentrations (2 × 10^9^, 5 × 10^8^, and 2 × 10^8^ colony‐forming units (CFUs) mL^−1^). The two former concentrations of AS1 resulted in 100% mortality in mice within 12 h of inoculation, with little difference observed between the *mcr‐3*‐positive AS1‐infected and *mcr‐3*‐negative AS1‐infected groups (Figure S2A,B, Supporting Information). Thus, the survival curves indicated that survival rate directly correlates with the dose of AS1 (Figure S2A,B, Supporting Information). We hypothesized that a high concentration of bacteria stimulated a strong inflammatory response, causing severe sepsis and death that occurred too rapidly to observe differences in virulence between these strains. Therefore, we used a bacterial concentration of 2 × 10^8^ CFU mL^−1^ to examine whether the acquisition of *mcr‐3* by AS1 had a significant impact on virulence. Compared with AS1 harboring the empty pHSG299 vector, mice challenged with *mcr‐3*‐positive AS1 exhibited much higher mortality rates (Figure S2C, Supporting Information; two‐tailed unpaired *t*‐test; *P* < 0.001). In mice infected with AS1, we observed acute inflammation in the lung regardless of the presence of *mcr‐3*, with infiltration of numerous polymorphonuclear cells as well as consolidation. Severe lung damage was observed in mice infected with *mcr‐3*‐positive AS1, and >60% of these mice also demonstrated pneumorrhagia. In contrast, mice infected with *mcr‐3*‐negative AS1 showed only minor inflammation and thickening of diaphragmatic walls in the lungs (**Figure** **2A**). We also observed low‐level inflammation and minor pathological changes in spleen, liver, and kidney (Figure S3, Supporting Information).

Lipid A modification reportedly decreases the activation of the host innate immune system.^[^ ^20^ ^]^ Therefore, we injected mice with bacteria at the concentration of 5 × 10^7^ CFU mL^−1^ and evaluated symptoms of infection in the host using a standard sepsis model in which clinical scores and body temperatures of mice were used to confirm infection (Figure 2B,C). Infection with the *mcr‐3*‐positive AS1 strain resulted in more severe symptoms (appearance, behavior, clinical signs, hydration status, and body temperature) in mice at 6 h postinfection compared with those animals infected with the *mcr‐3*‐negative strain (Table S8, Supporting Information). At 3 h postinfection, a significantly greater bacterial burden was observed in the blood of *mcr‐3*‐positive AS1‐infected mice compared with *mcr‐3*‐negative AS1‐infected mice (Figure 2D; two‐tailed unpaired *t*‐test; *P* < 0.05). A significant difference (Figure 2E; two‐tailed unpaired *t*‐test; *P* < 0.05) in monocyte and neutrophil counts examined by routine blood test was observed between mice infected with the *mcr‐3*‐positive strain and the phosphate‐buffered saline (PBS)‐infected control group, while no significant difference was observed between the *mcr‐3*‐negative AS1‐infected mice and the control. Increased levels of interleukin (IL)‐6, tumor necrosis factor (TNF)‐*α*, and IL‐1*β* were also observed (Figure 2F) in the *mcr‐3*‐positive AS1‐infected group compared with the *mcr‐3*‐negative AS1‐infected mice. However, the results indicated that mice infected with either strain demonstrated sepsis. In the organs of *mcr‐3*‐positive AS1‐infected mice, IL‐1*β* and TNF‐*α* mRNA levels were increased at 12 h postinfection (Figure S4, Supporting Information) compared with the control, especially in liver and lung tissues. In comparison, organ cytokine expression levels in mice infected with *mcr‐3*‐negative AS1 increased in the early stages of infection but decreased throughout the experimental period (Figure S4, Supporting Information).

In addition to combatting *Aeromonas* species, colistin is a critically important antibiotic for the treatment of infections caused by Enterobacteriaceae species such as *E. coli*. To examine whether *mcr‐3* affects the virulence of Enterobacteriaceae, we transformed *E. coli* DH5*α* with pHSG299‐*mcr‐3*. An eightfold increase in colistin MIC was observed for the transformants compared with the wild‐type strain (Figure 1A), with acquisition of pHSG299‐*mcr‐3* also resulting in a higher mortality rate compared with *mcr‐3*‐negative strains that were as similar as AS1 (Figure S2D, Supporting Information).

### 2.4 Expression of MCR‐3 Increased Bacterial Accumulation In Vivo

**Figure 3:**
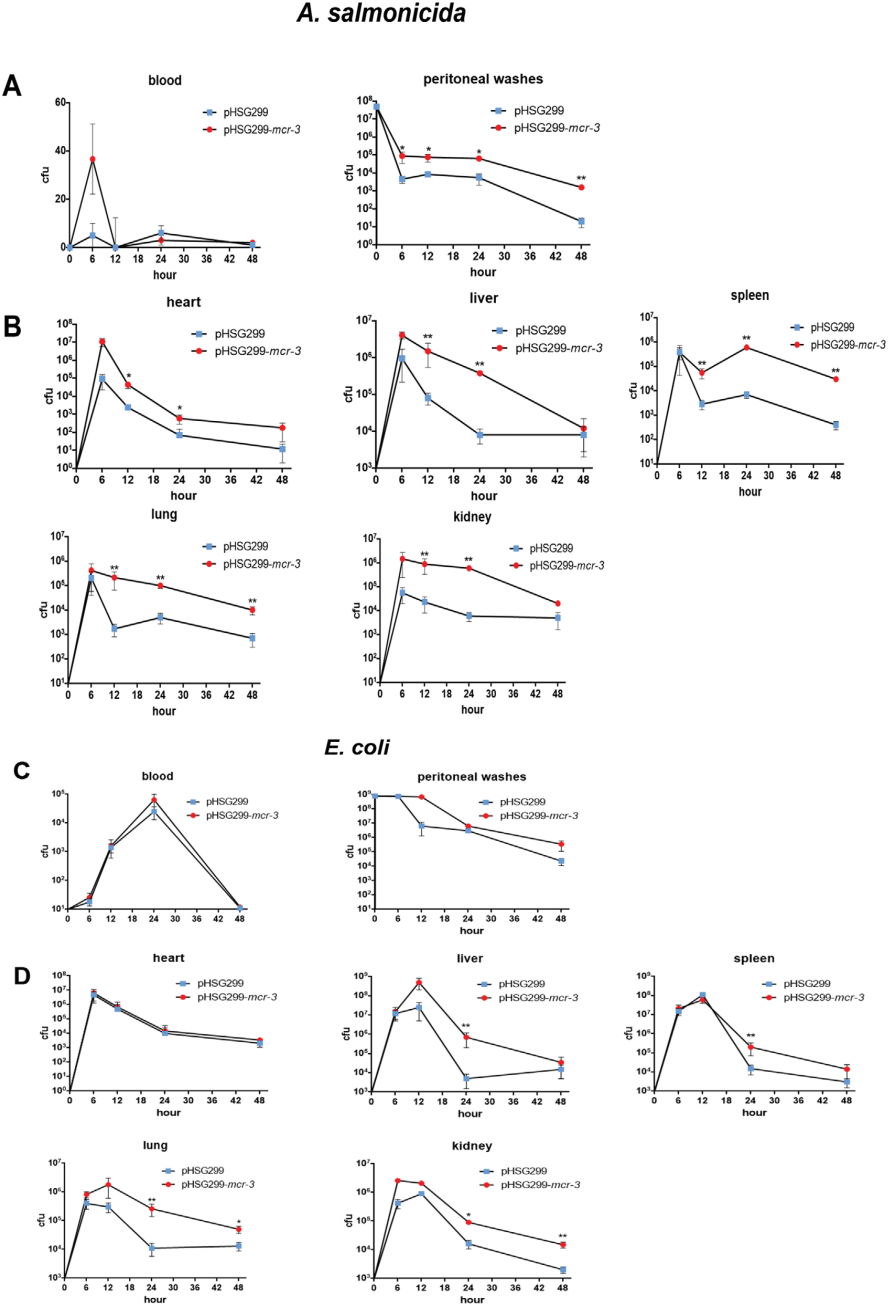
Bacterial loads in the tissues of infected mice. A,C) Bacterial counts per 100 µL of blood and peritoneal washes (three mice were tested at each time point). B,D) Bacterial counts in 100 µL of tissue homogenate (three mice were tested at each time point). Results indicated the mean ± SEM. Statistical significance was assessed by two‐tailed unpaired *t*‐test; ^**^ *P* < 0.01 and ^*^ *P* < 0.05.

Subsequently, we compared the ability of the *mcr‐3*‐positive/negative AS1 strains to cause infection by performing a time‐course bacterial scavenging experiment. Compared with the *mcr‐3*‐positive strain, the *mcr‐3*‐negative strain was cleared more quickly from peritoneal washes and remained at lower levels in the blood of infected animals (Figure S5A, Supporting Information; **Figure** **3A**). This suggested that MCR‐3‐induced modification of lipid A contributes to bacterial persistence in vivo, allowing for a greater number of bacteria to survive in the peritoneum. Following bloodstream infection, *mcr‐3*‐positive AS1 invaded the heart, liver, spleen, lung, and kidney tissues of mice (Figure S5A, Supporting Information; Figure 3B), which would ultimately lead to multiple organ failure. In comparison, the *mcr‐3*‐minus strain was quickly eliminated from all organs. To investigate whether MCR‐3‐mediated LPS modification also affected the clearance of clinically important Enterobacteriaceae, persistance of DH5*α* in vivo was evaluated using the standard sepsis model (Figure 2G–I; Table S9, Supporting Information). Compared with *E. coli* DH5*α* harboring empty pHSG299 vector, the *mcr‐3*‐positive *E. coli* strains demonstrated higher persistence rates in mice (Figure S5B, Supporting Information; Figure 3C,D).

### 2.5 Expression of MCR‐3 Causes More Severe Hypoxia in Tissues

**Figure 4:**
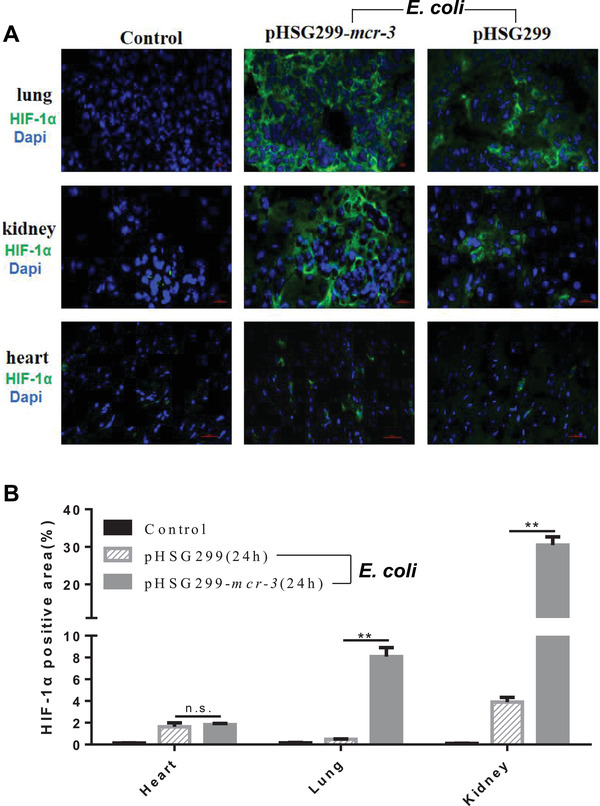
A) Cross‐sections of lung, kidney, and heart tissues collected 24 h postinfection with *mcr‐3*‐positive or *mcr‐3*‐negative *E. coli*. Sections were immunostained with anti‐HIF‐1*α* (green fluorescence indicates areas of hypoxia, bar = 10 µm). B) Quantification of hypoxic areas following immunostaining of tissue sections (calculated from at least five fields per slide, two‐tailed unpaired *t*‐test; ^**^ *P* < 0.01).

Increased expression of hypoxia‐inducible factor‐1*α* (HIF‐1*α*), which is a hallmark for tissue hypoxia, and which plays a key role in modulating innate immune cell function, has been observed in cells and tissues infected with bacterial pathogens.^[^ ^32^, ^33^ ^]^ Therefore, we tested the HIF‐1*α* protein of tissues in several important organs by immunofluorescent staining to analyze the hypoxia level. In our model, mice infected with either *mcr‐3*‐positive or *mcr‐3*‐negative *E. coli* showed signs of hypoxia in lung, kidney, and heart tissues (**Figure** **4A**). However, HIF‐1*α* positive area was increased in lung and kidney of mice infected with *mcr‐3*‐positive *E. coli* compared with those infected with the *mcr‐3*‐negative strain, while there was no significant difference in HIF‐1*α* positive area in the heart between the two groups (Figure 4A,B; two‐tailed unpaired *t*‐test; *P* < 0.01). This data coincided with the results of bacterial clearance assays.

### 2.6 Decoration Mediated by MCR‐3 Effectively Protects Bacteria from the Phagocytosis of Host Immunocytes

To explain the differences in clearance rate of the *mcr‐3*‐positive/negative bacteria in mice, *E. coli* was used in vivo to examine the influence of MCR‐3 expression on phagocytosis efficiency by phagocytes.

**Figure 5:**
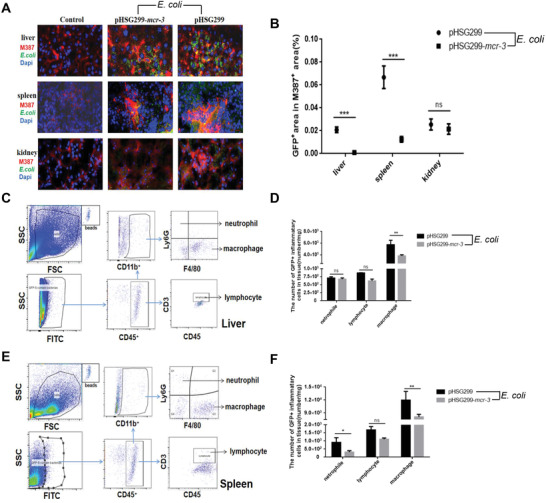
A) Cross‐sections of liver, spleen, and kidney tissues 24 h postinfection with *mcr‐3*‐positive or *mcr‐3*‐negative *E. coli*. Sections were immunostained with anti‐MAC387 (red fluorescence, monocytes/macrophages) and anti‐*E. coli* (green fluorescence, *E. coli*, bar = 10 µm). B) Percentages of red and green double positive area in red area were quantificated in tissue sections, indicative of macrophages containing phagocytosed *E. coli* (calculated from at least five fields per slide; ^***^ *P* < 0.001). C) Quantitative analysis of absolute counts of liver inflammatory cells by flow cytometry (*n* = 3) following infection with *mcr‐3*‐positive or *mcr‐3*‐negative *E. coli* harboring a GFP fluorescent label. D) Absolute counts of neutrophils, lymphocytes, and macrophages in liver containing phagocytosed fluorescently tagged *E. coli* at 24 h postinfection (*n* = 3, ^**^ *P* < 0.01). E) Quantitative analysis of percentages and absolute counts of inflammatory cells harboring GFP‐tagged *mcr‐3*‐positive or *mcr‐3*‐negative *E. coli* in the spleen, determined by flow cytometry (*n* = 3). F) Absolute counts of neutrophils, lymphocytes, and macrophages in spleen tissues containing phagocytosed fluorescently tagged *E. coli* at 24 h postinfection (*n* = 3, ^*^ *P* < 0.05, ^**^ *P* < 0.01). Statistical significance was assessed by two‐tailed unpaired *t*‐test.

To compare the phagocytosis efficiency of *mcr‐3*‐positive/negative bacteria by macrophages in vivo, we determined the proportion of all macrophages versus those containing phagocytosed *E. coli* in mouse tissues following infection. Protein MAC387 (macrophage marker) and *E. coli* were labeled with red and green fluorescence, respectively, in tissue sections. The percentages of red and green double positive area in red area were quantitatively identify macrophages containing phagocytosed *E. coli*. Analysis of tissue sections revealed a significantly higher proportion of areas containing double red and green fluorescence in liver and spleen tissues from mice infected with *mcr‐3*‐negative *E. coli* compared with *mcr‐3*‐positive‐infected mice (two‐tailed unpaired *t*‐test; *P* < 0.01). No significant difference was detected in other tissues (**Figure** **5A**,B). Thus, modification of lipid A appeared to reduce the phagocytosis efficiency of macrophages in the liver and spleen at 24 h postinfection.

To further compare the phagocytosis efficiency of different inflammatory cells, we then quantified the numbers of macrophages, neutrophils, and lymphocytes containing phagocytosed *mcr*‐3‐positive/negative *E. coli* tagged with green fluorescent protein labels in liver and spleen by fluorescence‐activated cell sorting (FACS) analysis (Figure 5C,E). Absolute quantification revealed that the proportion of inflammatory cells containing *mcr‐3*‐positive *E. coli* was lower than that containing *mcr‐3*‐negative *E. coli* in the liver and spleen (Figure 5D,F). Together, these results suggested that MCR‐3 expression induced lipid A decoration may help the bacteria evade phagocytosis, especially by macrophages.

### 2.7 Expression of MCR‐3 in Bacteria Decreased Macrophages Activation

**Figure 6:**
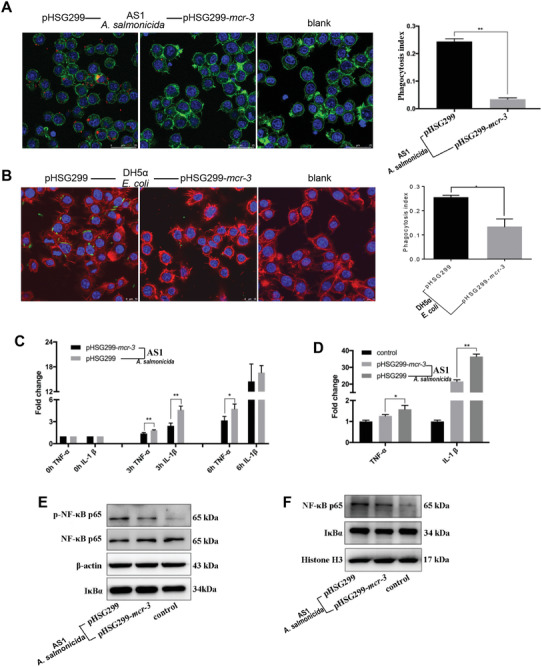
Compared with the wild‐type strain, *mcr‐3*‐positive AS1 suffered significantly less phagocytosis and showed reduced macrophage stimulation. A) Representative confocal laser scanning microscopy images showing phagocytosis of AS1 strains containing plasmids pHSG299 or pHSG299‐*mcr‐3*, along with uninfected controls. Calculation of the phagocytosis index (engulfed bacteria per macrophage, calculated from at least five fields per slide) based on confocal microscopy images (≥200 cells were scored per well). B) Representative confocal laser scanning microscopy images showing phagocytosis of DH5*α* strains containing plasmids pHSG299 or pHSG299‐*mcr‐3*, along with uninfected controls. Calculation of the phagocytosis index (engulfed bacteria per macrophage, calculated from at least five fields per slide) based on confocal microscopy images (≥200 cells were scored per well). C) Transcription of IL‐1*β* and TNF‐*α* in RAW264.7 macrophages stimulated with *mcr‐3*‐positive or *mcr‐3*‐negative AS1. D) Transcription of IL‐1*β* and TNF‐*α* in RAW264.7 macrophages stimulated with MCR‐3‐modified/unmodified LPS extracted from AS1. E) Immunoblotting analysis. Total expression of NF‐*κ*B‐pathway proteins (phosphorylated‐p65, p65, *β*‐actin, and I*κ*B*α*) in the lysates of RAW264.7 macrophages treated for 60 min with modified/unmodified LPS purified from *mcr‐3*‐positive/negative AS1. F) Nuclear protein expression in RAW264.7 macrophages treated for 60 min with MCR‐3‐modified/unmodified LPS from AS1. Histone was used as a control. Statistical significance was assessed by two‐tailed unpaired *t*‐test; ^**^ *P* < 0.01 and ^*^ *P* < 0.05. Error bars represent means ± SEM from triplicate wells.

To further verify that expression of MCR‐3 can help bacteria evade phagocytosis by macrophages in vitro and evaluate whether this immune evasion also occurred, we co‐cultured RAW264.7 macrophage cells with the *mcr‐3*‐positive/negative strains. Confocal microscopy and plate counting of intracellular bacteria were used to determine the phagocytosis index for each strain. Overall, the results were consistent with the bacterial survival rates observed in vivo. The phagocytosis index of RAW264.7 macrophage challenged with *mcr‐3*‐negative AS1 and DH5*α* was significantly higher than that of cells infected with *mcr‐3*‐positive strains (**Figure** **6A**,B; two‐tailed unpaired *t*‐test; *P* < 0.05). Phagocytosis index of *mcr‐3*‐positive/negative DH5*α* or AS1 obtained by plate counting exhibited the same tendency with those obtained via confocal microscopy (Figure S6, Supporting Information). This result indicated that lipid A modification mediated by *mcr‐3* affected the phagocytosis of macrophages and this was consistent with the results that *mcr‐3*‐positive bacteria failed to be cleared in vivo.

Therefore, to investigate whether MCR‐3‐modified LPS affects phagocyte activation, we examined the transcription of IL‐1*β* and TNF‐*α* in RAW264.7 cells stimulated with either live MCR‐3‐positive/negative AS1 or their purified modified/unmodified LPS (Figure 6C,D). Both *mcr‐3*‐positive AS1 and its modified LPS stimulated lower levels of cytokine transcription compared with the *mcr‐*3‐negative strain and its unmodified LPS, confirming that *mcr‐3*‐mediated LPS modification affects phagocyte activation.

The NF‐*κ*B signaling pathway is a classical pathway responsible for macrophage activation and immune factor transcription. Therefore, we assessed the activation of the NF‐*κ*B signaling cascade in macrophages challenged with LPS extracted from *mcr‐3*‐positive/negative AS1. In this pathway, the nuclear translocation of NF‐*κ*B is preceded by its phosphorylation and the subsequent degradation of nuclear factor of kappa light polypeptide gene enhancer in B‐cells inhibitor, alpha (I*κ*B*α*). Our results showed that the phosphorylated NF‐*κ*B p65 subunit was less abundant in macrophages challenged with MCR‐3‐modified LPS than in those infected with unmodified LPS (Figure 6E). To confirm the decreased translocation of NF‐*κ*B into the nuclei of cells challenged with modified LPS, we extracted the nuclear proteins of LPS‐stimulated cells and subjected them to western blotting. The results revealed that translocation of NF‐*κ*B from the cytoplasm into the nucleus of RAW264.7 cells decreased in cells stimulated with MCR‐3‐modified LPS compared with cells treated with unmodified LPS (Figure 6F). These results suggested that decreased NF‐*κ*B signaling activation, phagocytosis, and M1‐related cytokines in macrophages may be responsible for the greater abundance of *mcr‐3*‐positive bacteria in tissues compared with *mcr‐3*‐negtive bacteria (Figure 3). Moreover, high concentrations of *mcr‐3*‐positive bacteria in vivo induced severe hypoxia to cause tissue injury and inflammation despite their diminished capacity to activate NF‐*κ*B signaling compared with *mcr‐3*‐negtive bacteria.

## 3 Discussion

In hostile and uncertain environments, Gram‐negative organisms have evolved to overcome potential injury through modifying their LPS.^[^ ^20^ ^]^ Lipid A modification is carried out by specific enzymes (e.g., MCR variants) and is used by bacteria to protect against harmful compounds such as colistin.^[^ ^24^ ^]^ The MCR‐mediated low‐level colistin resistance of many Enterobacteriaceae is attributed to the singular addition of pEtN to lipid A.^[^ ^3^, ^34^ ^]^ However, for the first time, we have identified a dual mechanism, pEtN and l‐Ara4N, which might synergistically contribute to high‐level colistin resistance in *Aeromonas*species. The addition of l‐Ara4N to lipid A is carried out via the expression of the *arnBCADTEF* operon, which is usually controlled by a two‐component regulatory system.^[^ ^35^ ^]^ Compared with the increase in net charge from −1.5 to −1 mediated by the addition of pEtN, the addition of l‐Ara4N neutralizes the net negative charge of LPS,^[^ ^30^, ^36^ ^]^ leading to a significant decrease in binding affinity for colistin. However, neither the *arnBCADTEF* operon nor *mcr‐3* alone leads to high colistin resistance in *Aeromonas* species: only their co‐existence mediates high‐level resistance to this last‐resort antibiotic (Table S5, Supporting Information). It should be noted that some Enterobacteriaceae, including *K. pneumoniae* and *Salmonella* Typhimurium, possess a chromosomally encoded lipid A remodeling mechanism.^[^ ^30^ ^]^ It is not clear whether Enterobacteriaceae can synergistically use these mechanisms with *mcr* genes to produce high‐level colistin resistance phenotypes; however, the threat of *mcr*‐associated high‐level colistin resistance in various pathogens deserves significant attention.

Native lipid A, which is normally hexa‐acylated, *bis*‐phosphorylated, and conserved among Gram‐negative bacteria, has the strongest ability to activate TLR4.^[^ ^37^ ^]^ TLR4‐MD2 binds to the phosphate groups and acyl chains of lipid A via ionic and hydrophobic interactions, respectively.^[^ ^38^ ^]^ Most lipid A modifications interfere with TLR4‐MD2 recognition, protecting cells from phagocytosis and impacting the elimination of these invasive clinical pathogens.^[^ ^17^, ^22^, ^24^, ^38^, ^39^, ^40^ ^]^ Like the acyl‐chain and l‐Ara4N modifications of lipid A, the pEtN modification, which is predominantly regulated by a chromosomal two‐component system, can also lead to decreased stimulation of immune cells.^[^ ^20^, ^41^ ^]^ To the best of our knowledge, this is the first report of phagocytosis evasion mediated by plasmid‐borne *mcr‐3*, the protein product of which modifies the lipid A component of bacteria to interfere with TLR4‐MD2 recognition. In addition, this modification alters the net membrane charge, reducing the efficacy of host cationic antimicrobial peptides produced by the human body as part of the normal innate immune response. Whether other *mcr* genes (*mcr*‐1 to 10) also facilitate bacterial evasion of host phagocytosis will be verified in our subsequent studies. MCR‐mediated lipid A modification has several significant implications for clinical treatment, not only negating colistin treatment but also enhancing bacterial invasion and hindering pathogen clearance. Moreover, we observed that *A. salmonicida* harboring plasmid pHSG299‐*mcr‐3* was more stable in vivo than in vitro, which may be associated with the pressure imposed by the host innate immune system, with the same result observed for *mcr‐3*‐positive *E. coli*. It should be noted that *E. coli* was primarily used in the current study to prove this phenomenon in vivo due to the lack of an appropriate antibody for *A. salmonicida* using in flow cytometry and immunofluorescence assays. Collectively, the propensity for horizontal transfer and retention of *mcr* genes among various pathogens and the prolonged persistence of MCR‐producing pathogens in tissues raise additional challenges to the prevention and treatment of *mcr*‐positive bacterial infections. Therefore, these findings expand our current understanding of the pathogenesis associated with lipid A modifications.

Lipid A contains a molecular pattern that could be efficiently identified by macrophages, in consequence activating host defense responses. The liver, spleen, and lungs are the major organs defended by macrophages. Not surprisingly, our results showed that following infection with *mcr‐3*‐positive bacteria, immune activation and bacterial clearance by macrophages were significantly reduced in these organs. In addition to antibiotic resistance and innate immune evasion, MCR‐mediated lipid A modification was shown to have several other protective effects in bacteria, including enhanced resistance to lysozymes and hypertonic seawater.^[^ ^42^, ^43^ ^]^ We also observed the development of fimbria‐like structures on the outer membranes of *mcr‐3*‐positive *A. salmonicida* cells in the presence of high colistin concentrations (32 mg L^−1^; Figure 1D). This phenomenon suggested that MCR‐3 may have a similar mechanism to chromosomal phosphoethanolamine transferase EptC from *Campylobacter jejuni*, which has multifacet roles in lipid A modification and the induction of flagella.^[^ ^44^ ^]^ In general, the development of fimbria‐like structures places an additional burden on bacterial cells; however, these structures are critical for adaptation to hostile environments.^[^ ^45^ ^]^

The innate immune response, in which phagocytes play a major role, is the first line of defense against pathogen invasion. Here, we identified that the modification of lipid A by MCR‐3 decreased the activation of TLR4, resulting in reduced phagocyte stimulation. This result confirms that the extensive or long‐term use of colistin generates more severe consequences than previously recognition, not only facilitating the rapid spread of *mcr* genes among pathogens in both humans and animals, but also furnishing the *mcr*‐carrying pathogens with the ability to resist host innate immune defenses. Our findings may have important implications for the development of approaches targeting MCR‐3 to conquer the double‐barreled effects of colistin resistance and innate immune evasion. Applying various combinations of antimicrobials other than colistin and enhancing the host immune capacity might be a promising strategy.

## 4 Experimental Section

### Mice and Ethics Statement

Six‐week‐old female BALB/c mice were purchased from Beijing Vital River Laboratory Animal Technology Co., Ltd (Beijing, China). Mice were raised in a specific pathogen‐free experimental animal room for 1 week to prevent a stress reaction, before being used as a model to analyze bacterial infection and host immune responses. The mice were raised and handled in compliance with the Chinese laws and guidelines (protocol GKFCZ2001545), EU Directive 2010/63/EU for animal experiments, and the China Agricultural University regulations concerning the protection of animals used for scientific purposes (2010‐SYXK‐0037). Committee on Animal Welfare and Ethics in China Agricultural University approved the animal experiments (AW01201202‐2‐1).

### Bacterial Strains, Plasmid Construction, and Electroporation

Bacterial strains and plasmids used in this study were described in Table S3 in the Supporting Information. *E. coli* strain DH5*α* was purchased from Takara Bio (Shiga, Japan). *E. coli* DH5*α* were cultured at 37 °C, while *A. caviae* ZJ66‐1 and *A. salmonicida* AS1 were cultivated at 30 °C. The *arnBCADTEF* operon from strain AS1 and *mcr‐3* from *E. coli* WJ1^[^ ^14^ ^]^ were ligated into plasmid pHSG299 to generate plasmids pT and pHSG299‐*mcr‐3*, respectively (Figure S1A, Supporting Information). Plasmids pHSG299 and pHSG299‐*mcr‐3* were transformed into *A. salmonicida* AS1, and *E. coli* DH5*α*. Plasmid pT was transformed into *A. caviae* ZJ66‐1. All bacterial strains were cultured in Luria–Bertani (LB) medium supplemented with kanamycin (50 mg L^−1^) at 30 °C (*Aeromonas*) or 37 °C (Enterobacteriaceae).

### Macrophage Cell Line and Culture Conditions

The RAW264.7 murine macrophage cell line was obtained from the Cell Resource Center at the Institute of Basic Medical Sciences, CAMS/PUMC (Beijing, China) and cultured as previously described.^[^ ^46^ ^]^ Briefly, cells were cultured in high‐glucose Dulbecco's Modified Eagle's Medium (DMEM) containing 1% (v/v) penicillin/streptomycin and 10% (v/v) fetal bovine serum at 37 °C with 5% CO_2_.

### Whole‐Genome Sequencing

*A. salmonicida* strain AS1 was subjected to 150 bp paired‐end whole‐genome sequencing using the Illumina HiSeq X Ten platform (Annoroad, Beijing, China). Contigs were obtained by draft assembly using the CLC Genomics Workbench 9 (CLC Bio, Aarhus, Denmark).

### MIC, Growth Conditions, and Growth Curve Assays

The MICs of 12 different antibiotics (Table S2, Supporting Information) against *A. salmonicida* and its transformants were determined using a broth dilution method according to the Clinical and Laboratory Standards Institute document M45‐A2. All Enterobacteriaceae were cultivated at 37 °C, while the *Aeromonas* strains were cultured at 30 °C. For growth curve assays, *A. salmonicida* strains were suspended in brain heart infusion (BHI) broth containing colistin (concentrations ranging from 0 to 8 mg L^−1^), seeded into 96‐well plates at a concentration of 10^6^ cells per well in quadruple, and incubated at 37 °C in a microplate reader (Infinite 200 PRO, Tecan, Männedorf, Switzerland). Absorbance (OD_600_) values for each well were recorded every 30 min for 12 h.

### Isolation and Analysis of Lipid A

Bacterial lipid A was extracted using the hydroxide/isobutyric acid method. Briefly, bacteria were cultivated in 10 mL of LB broth at 30 °C to an OD_600_ = 0.9 (range: 0.8–1.0). Cells were collected by centrifugation and then washed with PBS. The cell pellet was then resuspended in 1 m ammonium hydroxide‐70% (v/v) isobutyric acid solution (5:3, v/v) to a final volume of 640 µL. Samples were boiled for 1 h and the supernatant was collected by centrifugation at 2000 × *g* for 20 min. An isochoric volume of sterile water was added to the supernatant, which was then frozen at −80 °C before being lyophilized. A 1 mL volume of methanol was used to wash the residue, and lipid A was extracted using extraction agent consisting of chloroform, methanol, and water (3:1:0.25, v/v).^[^ ^47^ ^]^ The extracted lipid A was analyzed using negative‐ion MALDI‐TOF mass spectrometry.

### Transmission Electron Microscope (TEM)

*A. salmonicida* stains harboring plasmids pHSG299 or pHSG299‐*mcr‐3* were cultured to log‐phase in tryptic soy broth (TSB) medium. Bacterial cells were then collected by centrifugation at 2000 × *g* and washed once with PBS. Cells were fixed in 2.5% (v/v) glutaraldehyde solution at 4 °C for 24 h. In preparation for TEM analysis, samples were embedded in Spur epoxy resin medium and polymerized at 70 °C for 3 days. Ultrathin sections were subsequently cut using a Leica Ultracut microtome (UC6i; Leica, Wetzlar, Germany) and stained with uranyl acetate and lead citrate in a Leica EM Stainer. The sections were examined using a JEOL JEM‐1230 transmission electron microscope (JEOL Ltd., Tokyo, Japan).

### Murine Infection Model

Bacterial strains were cultured on tryptic soy agar plates supplemented with kanamycin (50 mg L^−1^) at 30 °C for 24 h. A single clone was randomly selected from each strain and inoculated into TSB. When bacteria reached log‐phase growth, cells were collected by centrifugation for 10 min at 5000 × *g*. Cell pellets were washed once with PBS and then resuspended in the same buffer.

Prior to infection, mice were fasted for 12 h but given free access to water. The blank control group was intraperitoneally (i.p.) injected with 200 µL of PBS. Experimental and control groups were injected i.p. with *A. salmonicida* AS1 harboring pHSG299‐*mcr‐3* or pHSG299, respectively, at concentrations of 2 × 10^9^, 5 × 10^8^, or 2 × 10^8^ CFU mL^−1^ (0.2 mL per mouse) to determine the survival rates. Numbers of mice used at each step were outlined in Table S6 in the Supporting Information. At the time of sacrifice, internal tissues were collected and fixed in 4% (v/v) paraformaldehyde. The tissues were then washed with distilled water and dehydrated using an alcohol gradient. Xylene was then used to replace the alcohol and tissues were embedded in paraffin by wax immersion at 60 °C. Paraffin blocks were sectioned at a thickness of 4–7 µm, and sections were subsequently stained with hematoxylin and eosin and sealed with neutral gum.

Following survival assays, a new group of mice were injected with a sublethal dose of *A. salmonicida* AS1 (5 × 10^7^ CFU mL^−1^, 0.2 mL per mouse) to examine bacterial clearance rates.^[^ ^48^ ^]^ The infection status of each mouse was then evaluated according to the standard criteria used in this research for defining sepsis. The number of mice used is outlined in Table S6 in the Supporting Information. Two groups (*n* = 6 mice per group) of mice were sacrificed at 0 h postinfection for use as the blank control. At 3, 6, 12, and 24 h postinfection, the skin temperatures of the alive mice were recorded using an infrared thermometer (OMRON, Kyoto‐fu, Japan), and the clinical scores were assessed as described in Table S7 in the Supporting Information.^[^ ^49^ ^]^ At each time point, three mice from each group were randomly sacrificed and blood, peritoneal washes, and internal tissues were harvested. Plate counts were conducted for each of the samples to assess bacterial burden. At the same time, samples were serially diluted and 10 µL of each was spotted onto LB agar containing kanamycin (50 µg mL^−1^), as described previously.^[^ ^50^ ^]^ Plates were then incubated overnight at the appropriate temperature before being photographed for CFU counts. Serum was also collected from blood samples and used for routine blood tests. RNA was extracted from each tissue sample and used to examine transcription levels of IL‐1*β* and TNF‐*α* by quantitative reverse‐transcriptase polymerase chain reaction (qRT‐PCR) analysis. Enzyme‐linked immunosorbent assay (ELISA) analysis was used to measure the concentrations of IL‐1*β*, IL‐6, and TNF‐*α* in serum using an IL‐1*β* Mouse ELISA Kit, an IL‐6 Mouse ELISA Kit, and a TNF‐*α* Mouse ELISA Kit (Thermo Fisher Scientific, Waltham, MA, USA) according to the manufacturer's instructions.

Survival and bacterial clearance assays were also conducted as described above for *E. coli*. For the survival curve assays, *E. coli* was injected i.p. at concentrations of 8 × 10^8^ CFU mL^−1^ (0.2 mL per mouse).

### Histology and Imaging

Immunofluorescence was performed as described previously.^[^ ^51^ ^]^ Following administration of anesthesia, mice were perfused with PBS through the left ventricle. The visceral organs (lung, spleen, kidney, and liver) were then removed and processed for cryosectioning. Immunofluorescence was performed using antibodies against HIF‐1*α*, *E. coli*, and MAC387 (all antibodies were purchased form Abcam, Cambridge, MA, USA). Sections were permeabilized in 0.3% (v/v) Triton X‐100 in PBS and blocked with Protein Block (DAKO, Glostrup, Denmark) for 1 h at room temperature. The sections were incubated with a primary antibody mixed with diluting solution (Beyotime Biotechnology, Shanghai, China) overnight at 4 °C and then detected using secondary antibodies conjugated to Alexa 568 (Invitrogen, Carlsbad, CA, USA) or 488 (Abcam, Cambridge, MA, USA). Tissues were visualized using a Nikon 80i microscope, and images were acquired using DS‐cooled camera and NIS‐Elements Br 3.0 software (Melville, NY, USA). Hypoxia and phagocytosis were evaluated by calculating the sizes of the areas of green (HIF‐1*α*‐positive area) and yellow (overlap area of *E. coli* and MAC387) staining in comparison to the total tissue area using an Image Pro Plus quantitative automatic program (Media Cybernetics, USA). For all semiquantitative morphological analyses, at least three fields were counted from each slide.

### Flow Cytometry

Following administration of anesthesia, three mice from each group were randomly sacrificed and their blood, livers, and spleens were harvested. Liver and spleen tissues were dissected and ground separately before being digested with 2 mL of collagenase types II and IV (2.5 U mL^−1^; Sangon Biotech, Shanghai, China), respectively, in PBS with 10 × 10^−3^ m CaCl_2_ at 37 °C for 30 min. After washing, the tissues were separately passed through a 70 µm strainer (Becton Dickenson (BD), Franklin Lakes, NJ, USA) and washed with PBS as described previously.^[^ ^51^ ^]^ The resulting single‐cell extracts and blood samples were then lysed using BD FACS Lysing Solution. Cells were collected by centrifugation at 300 × *g* for 5 min and then incubated in PBS containing 2 × 10^−3^ m ethylenediaminetetraacetic acid, 2% (v/v) fetal bovine serum, and primary antibodies for 30 min at 4 °C. The cells were then resuspended at ≈1 × 10^7^ cells mL^−1^ and analyzed using a BD FACSCanto II flow cytometer. The antibodies used in this investigation included anti‐CD11b‐APC, anti‐Ly6G‐BV421, anti‐F4/80‐PE, anti‐CD3e‐BV510, and anti‐CD45‐Percp. The cells were counted using CountBright Absolute Counting Beads (Invitrogen). Staining was performed in accordance with the manufacturer's protocol.

### Macrophage Stimulation by *mcr‐3*‐Positive and *mcr‐3*‐Negative *A. salmonicida* AS1

Bacteria were cultured overnight in BHI broth supplemented with kanamycin (50 mg L^−1^) before being washed with PBS. RAW264.7 cells were seeded in 6‐well plates at a concentration of 3 × 10^6^ cells per well and then cultured for 6 h to allow attachment. Bacterial strains were added to triplicate wells at a multiplicity of infection (MOI) of 0.01 (3 × 10^6^ RAW264.7 cells, 3 × 10^4^ bacterial cells per well) and plates were incubated for 3 and 6 h. Triplicate wells of RAW264.7 without bacteria served as the negative controls.

Cytokine (IL‐1*β* and TNF‐*α*) transcription in infected cells was assessed by qRT‐PCR analysis. Experiments were performed in triplicate, and fold‐change values were determined after normalization of gene expression to that of the glyceraldehyde‐3‐phosphate dehydrogenase gene (GAPDH) using the comparative threshold method.^[^ ^52^ ^]^

### Macrophage Stimulation by LPS from *mcr‐3*‐Positive and *mcr‐3*‐Negative *A. salmonicida* AS1

Bacteria were cultured overnight in BHI broth supplemented with kanamycin (50 mg L^−1^) and then washed with PBS. LPS was extracted from bacteria using an LPS Extraction Kit (iNtRON Biotechnology, Burlington, MA, USA) according to the manufacturer's instructions. The extracted LPS was quantified by Purpald assay as described previously.^[^ ^53^ ^]^ MCR‐3‐modified or unmodified LPS (1 µg per well) was then added to triplicate wells containing RAW264.7 seeded in 6‐well plates at a concentration of 3 × 10^6^ cells per well and incubated at 37 °C with 5% CO_2_ for 18 h. RAW264.7 cells without LPS served as the negative controls. Cytokine (IL‐1*β* and TNF‐*α*) transcription in the cells was then detected by qRT‐PCR analysis as described above.

### Phagocytosis Assay

Strains were cultured overnight in BHI broth supplemented with kanamycin (50 mg L^−1^), washed with PBS. Murine RAW264.7 cells were seeded into 24‐well plates at a concentration of 1 × 10^6^ cells per well and cultured in Dulbecco’s modified eagle’s medium (DMEM) with 1% fetal bovine serum (FBS) at 37 °C for 6 h to allow attachment. The medium was then replaced with 1 mL of fresh DMEM (1% FBS) containing *mcr‐3*‐positive or *mcr‐3*‐negative *A. salmonicida* (in triplicate) at an MOI of 10 (3 × 10^6^ RAW264.7 cells, 3 × 10^7^ bacterial cells per well). Murine RAW264.7 cells were treated with *E. coli* at a MOI of 1. To examine phagocytosis, cells were incubated with bacteria for 60 min before the addition of streptomycin (1 g L^−1^) for 60 min to kill extracellular bacteria. Following incubation, cells were washed twice with PBS, and 0.5 mL of 1% (v/v) Triton X‐100 solution was added to each well. The number of intracellular bacteria was determined by quantitatively plating the lysates onto BHI agar. The phagocytosis index was determined based on the number of engulfed cells per macrophage as described previously.^[^ ^49^ ^]^ Statistical significance was assessed by *t*‐test. A *P*‐value of <0.05 was considered statistically significant.

### Microscopy

RAW264.7 cells were cultured with the *A. salmonicida* and *E. coli* strains as described for the phagocytosis assay. *A. salmonicida* was dyed by pHrodo (Invitrogen) according to manufacturer's instructions. GFP‐carrying *E. coli* was used in this assay. Following elimination of extracellular bacteria, 4',6‐diamidino‐2‐phenylindole (DAPI, Beyotime Biotechnology) and rhodamine phalloidin (Invitrogen) were used to stain the cell nuclei and *β*‐actin, respectively. Phagocytosis images were obtained by Leica TCS‐SP8 confocal microscope. The phagocytosis index was then determined based on the number of engulfed cells per macrophage, as described previously.^[^ ^54^ ^]^ At least 200 cells per well were scored.

### Immunoblotting

RAW264.7 cells were seeded into 6‐well plates at a concentration of 4 × 10^6^ cells per well. MCR‐3‐modified or unmodified LPS (2 µg per well) was added to each well and plates were incubated at 37 °C with 5% CO_2_ for 1 h. RAW264.7 cells without LPS served as the negative control. Whole‐cell lysates were prepared using RIPA Lysis Buffer (Beyotime Biotechnology), and 120 µg of total protein per sample was fractionated by 10% sodium dodecyl sulfate‐polyacrylamide gel electrophoresis. The gels were then electrophoretically transferred to polyvinylidene fluoride (PVDF) membranes (Solarbio, Beijing, China). Transferred proteins were detected using specific antibodies against the phosphorylated and nonphosphorylated forms of NF‐*κ*B p65, I*κ*B*α*, *β*‐actin, and Histone H3 (obtained from Beyotime Biotechnology and Cell Signal Technology, Danvers, MA, USA) according to the manufacturer's instructions. Membranes were developed using horseradish peroxidase‐labeled goat antirabbit/mouse IgG (H+L) secondary antibodies (Beyotime Biotechnology) and then visualized using an enhanced ECL Kit (ABclonal, Woburn, MA, USA).

### qRT‐PCR Analysis

RNA was extracted from heart, liver, spleen, lung, kidney, and brain tissues from experimental mice using an RNeasy Mini Kit (QIAGEN, Hilden, Germany). Reverse transcription of no more than 80 ng of RNA per sample was performed using a PrimeScript RT Reagent Kit with gDNA Eraser (Takara Bio). The resulting cDNA was diluted to a standard concentration and used as template for qRT‐PCR analysis using an Applied Biosystems 7500 Real‐Time PCR System (Thermo Fisher Scientific) with SYBR green and primers targeting TNF‐*α*, IL‐1*β*, and GAPDH (internal control). The thermal cycler parameters consisted of 95 °C for 3 min, followed by 40 cycles of 95 °C for 30 s, 60 °C for 30 s, and 72 °C for 30 s. Bacterial RNA was extracted and analyzed using the same method, with the addition of lysozyme prior to extraction to lyse the bacterial cells. The expression of *arnT* and *mcr‐3* was examined using this method, with the 16S rRNA gene used as an internal control. All primers used for qRT‐PCR analyses are described in Table S4 in the Supporting Information.

### Antimicrobial Peptide Susceptibility Assay

Bacteria were cultured at 30 °C in 5 mL of LB medium to an OD_600_ = 0.6 before being harvested at 2500 × *g* for 20 min. Cell pellets were washed with PBS and then resuspended to a concentration of ≈10^6^ CFU mL^−1^ in a solution consisting of 10 × 10^−3^ m PBS (pH 6.5), 1% TSB (Oxoid, Nepean, Canada), and 100 × 10^−3^ m NaCl. Aliquots (5 µL) of this suspension were mixed in 1.5 mL microcentrifuge tubes with various concentrations of antimicrobial peptide (LL‐37).^[^ ^55^ ^]^ In all cases, the final volume was 30 µL. After 1 h of incubation at 30 °C, the contents of the tubes were plated onto LB agar plates. Colony counts were determined, and the results were expressed as percentages of the colony counts of samples not exposed to antibacterial agents. All experiments were performed in duplicate on at least four independent occasions.

### Statistical Analysis

Results were expressed as mean ± standard error of mean (SEM). Sample size (*n*) for each statistical analysis was three samples per group (indicated in each figure). Immunostaining of tissue sections and Confocol sections were calculated from at least five fields per slide. The Graphpad prism 6 software was used for graph plotting. Statistical analysis was performed through two‐tailed unpaired *t*‐test. *P* < 0.05 was considered statistically significant.

### Accession Number

The sequences of *A. salmonicida* AS1 have been deposited in GenBank under BioProject PRJNA734781.

## Conflict of Interest

The authors declare no conflict of interest.

## Author Contributions

W.Y., Z.L., Y.D., L.Q., and Y.S. contributed equally to this work. J.S., G.F.G., Y.W., and T.R.W. designed the study. W.Y., Z.L., and Y.W. wrote the manuscript. W.Y., Z.L., Y.S., and L.Q. performed the investigation of colistin resistant mechanism. W.Y., Z.L., Y.D., and W.L. constructed the sepsis model. Z.L., L.Q., and Y.W. performed macrophage experiments. Y.D., C.D., and L.Q. performed the immunoblotting. J.S., G.F.G., T.R.W., Y.S., J.L., H.Y., D.L., and R.Z. participated in editing the manuscript.

## Supporting information

Supporting Information

## Data Availability

Genome sequencing data of *A. salmonicida AS1* have been deposited into Sequence Read Archive (SRA) database in NCBI under BioProject PRJNA734781. Other data that support the findings of this study are available from the corresponding author upon reasonable request.
