# Supplementary material for: Mobile Colistin Resistance Enzyme MCR‐3 Facilitates Bacterial Evasion of Host Phagocytosis
Source: Adv Sci (Weinh). 2021 Jul 29;8(18):2101336. doi: 10.1002/advs.202101336 (PMC8456205; doi:10.1002/advs.202101336)
Supplement: Supplementary file 1 — Supporting Information [file ADVS-8-2101336-s001.pdf]

© 2021 Wiley-VCH GmbH

ADVANCED SCIENCE
Open Access

Supporting Information

for *Adv. Sci.*, DOI: 10.1002/advs.202101336

Mobile colistin resistance determinant MCR-3 facilitates bacterial evasion of host phagocytosis

Wenjuan Yin#, Zhuoren Ling#, Yanjun Dong#, Lu Qiao#, Yingbo Shen#, Zhihai Liu, Yifan Wu, Wan. Li, Rong Zhang, Timothy Rutland Walsh, Chongshan Dai, Juan li, Hui Yang, Dejun Liu, Yang Wang, George Fu Gao*, Jianzhong Shen**

Table S1 Antibiotic Resistance Genes Identified in *Aeromonas salmonicida* Strain AS1

Gene	*ampH*	*bla*$_{FOX-4}$	*imiH*	*vatF*	*tet*(34)	*dfrA3*
(identity)	(85%)	(88%)	(91%)	(71%)	(68%)	(69%)
	beta-lactamase	beta-lactamase	beta-lactamase	macrolide	tetracycline	trimethoprim

Table S2 MICs (mg/L) of Tested Antibiotics Against *mcr-3*-Positive/Negative AS1 Strains

Strain / Antibiotics	pHSG299-*mcr-3* *Aeromonas salmonicida*	pHSG299 *Aeromonas salmonicida*
Colistin	64	1
polymycin	32	1
ceftazidime	0.0625	0.0625
gentamicin	0.5	0.25
amoxicillin-clavulanic acid	128/64	128/64
chloramphenicol	1	1
tetracycline	0.5	1
imipenem	4	1
meropenem	1	0.5
aztreonam	8	4
ciprofloxacin	0.015	0.015
trimethoprim-sulfamethoxazole	0.21/4	0.21/4

Table S3 Relevant Bacterial Strains and Plasmids Used in This Study

Strain or plasmid	Description	Source or Ref.
A. salmonicida		
AS1	Wild type (*Aeromonas salmonicida)*	[1]
ZJ66-1	Wild type (*Aeromonas caviae*)	[2]
E. coli		
DH5α	Wild type	Takara
Plasmids		
pHSG299	Cloning vector, Km^r	Takara
pHSG299-*mcr-3*	Cloning vector pHSG299 harboring *mcr-3* gene	This work
pT	Cloning vector pHSG299 harboring *arnBCADTEF* operon	This work

Table S4 Primers Used for qRT-PCR Analyses

Name	Sequencing (5'-3')	Length(bp)	Reference
mcr-3qr-F	ACCTCCAGCGTGAGATTGTTCCA	169	[3]
mcr-3qr-R	GCGGTTTCACCAACGACCAGAA		
16S-qr-F	CGGTGAATACGTTC(C/T)CGG	143	[4]
16S-qr-R	GG(A/T)TACCTTGTTACGACTT		
arnT-qr-F	ACTCAAGGAGGGGAAAGCAC	195	This study
arnT-qr-R	AACTGGATGAAACCGAGCAG		
GADPH-F	TGCCCCCATGTTTGTGATG	151	This study
GADPH-R	TGTGGTCATGAGCCCTTCC		
IL-1β-F	AACCTTTGACCTGGGCTGTC	144	This study
IL-1β-R	AAGGTCCACGGGAAAGACAC		
TNF-α-F	AGCCCCCAGTCTGTATCCTT	144	This study
TNF-α-R	TGATGGTGGTGCATGAGAGG		

Table S5 Dissemination of *mcr-3* Among Various *Aeromonas* Strains

Strain	Colistin MIC (mg/L)	*mcr*	*arnT*	genus	source
AS1	1	-	+	*Aeromonas salmonicida*	This study
AS1+*mcr-3*	64	*mcr-3*	+	*Aeromonas salmonicida*	This study
ZJ66-1	1	*mcr-3*	-	*Aeromonas caviae*	[2]
ZJ66-1 + *arnBCADTEF*	32	*mcr-3*	+	*Aeromonas caviae*	This study
101	64	*mcr-3*	+	*Aeromonas veronii*	Storage in our laboratory
82	64	*mcr-7*	+	*Aeromonas veronii*	Storage in our laboratory
Z5-5	32	*mcr-3*	+	*Aeromonas salmonicida*	[2]
235	64	*mcr-3*	+	*Aeromonas jandaei*	Storage in our laboratory
Z9-6	32	*mcr-3*	+	*Aeromonas allosaccharophila*	[2]
172	2	*mcr-3*	-	*Aeromonas veronii*	[1]
I47	8	*mcr-5*	-	*Aeromonas hydrophila*	[5]

Table S6. Numbers of Mice Used in the Different Steps

Time (hours after i.p.)	0	3/6[3]	6/12	12/24	24/48
colony count and numeration of leukocyte	3（blank control）	12[1]	12	12	12
sampling	blood/peritoneal washes /organs				
ELISA detection	3（blank control）	0	12		
sampling	serum				
survival line（AS1, DH5α）	120[2]				

[1,2]A total of 12 mice were divided into two groups and injected intraperitoneally with *A. salmonicida* AS1, *E. coli* DH5α. Each group was then equally divided and injected intraperitoneally with the same strain with/without *mcr-3*.
[3]Mice injected intraperitoneally with strain AS1 and DH5α were observed for 48 h.

Table S7 Standard Used for Assigning Clinical Scores [6]

Observing targets	condition	score
appearance	normal	0
	lack of grooming	1
	piloerection	2
	hunched up	3
	above and eyes half closed	4
behaviour - unprovoked	normal	0
	minor changes	1
	less mobil and flock together	2
	restless or very still	3
behaviour - provoked	responsive and alert	0
	unresponsive and not alert	3
clinical signs	normal respiratory rate	0
	slight changes	1
	decreased rate with abdominal breathing	2
	tachypnea and cyanosis	3
hydration status	normal	0
	dehydrated	5

Table S8 Clinical Scores of Mice Infected With *mcr-3*-Positive/Negative *Aeromonas salmonicida* Strains

clinical signs	0h	S[1]-3h	D[2]-3h	S-6h	D-6h	S-12h	D-12h	S-24h	D-24h
appearance	0	1	1	4	4	4	4	0	0
behaviour - unprovoked	0	1	1	2	2	2	2	0	0
behaviour - provoked	0	3	3	3	3	3	3	0	0
clinical signs	0	2	1	3	2	1	1	0	0
hydration status	0	0	0	0	0	5	0	0	0
Total	0	7	6	12	11	15	10	0	0

[1]S, mice infected with *mcr-3*-positive *A. salmonicida* strain AS1
[2]D, mice infected with *mcr-3*-negative *A. salmonicida* AS1

Table S9 Clinical Scores of Mice Infected With *mcr-3*-Positive/Negative *E. coli* Strains

clinical signs	0h	S1-3h	D2-3h	S-6h	D-6h	S-12h	D-12h	S-24h	D-24h	S-48h	D-48h
appearance	0	1	1	1	1	1	2	3	1	1	1
behaviour - unprovoked	0	2	2	2	2	2	2	2	2	1	1
behaviour - provoked	0	3	3	3	3	3	3	3	3	0	0
clinical signs	0	3	1	3	2	3	3	2	2	2	2
hydration status	0	0	0	5	0	5	2	2	0	1	1
Total	0	9	7	14	8	14	12	12	8	5	5

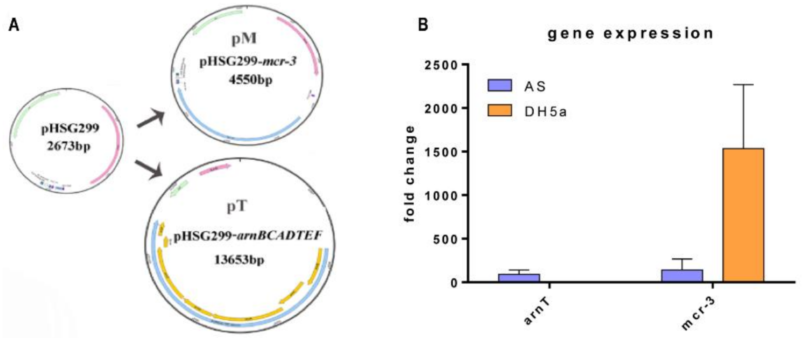

Figure S1 (A) Diagrams of the pHSG299-*mcr-3* and pT plasmid vectors. (B) *mcr-3* and *arnT* gene expression in *E. coli* and *A. salmonicida*, respectively.

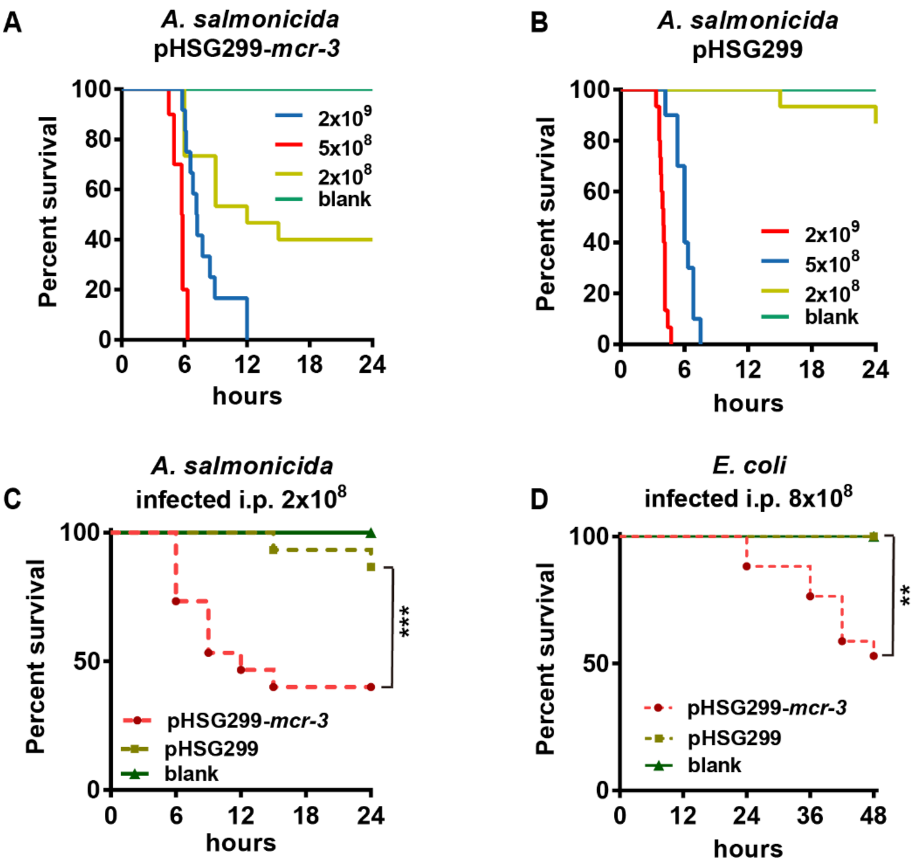

Figure S2 (A, B) Survival curves of mice injected intraperitoneally with 2×10^9, 5×10^8, or 2×10^8 CFU/mL *mcr-3*-positive/negative A. salmonicida strain AS1 (n = 15 mice/group). (C) Comparison of the survival of mice injected intraperitoneally with *mcr-3*-positive/negative AS1 at a concentration of 2×10^8 CFU/mL (n = 15 mice/group; ***P < 0.001). (D) Survival curves of mice injected intraperitoneally with *E. coli* harboring pHSG299 or pHSG299-*mcr-3* at concentrations of 8×10^8 CFU/mL (n = 15 mice/group; **P < 0.01).

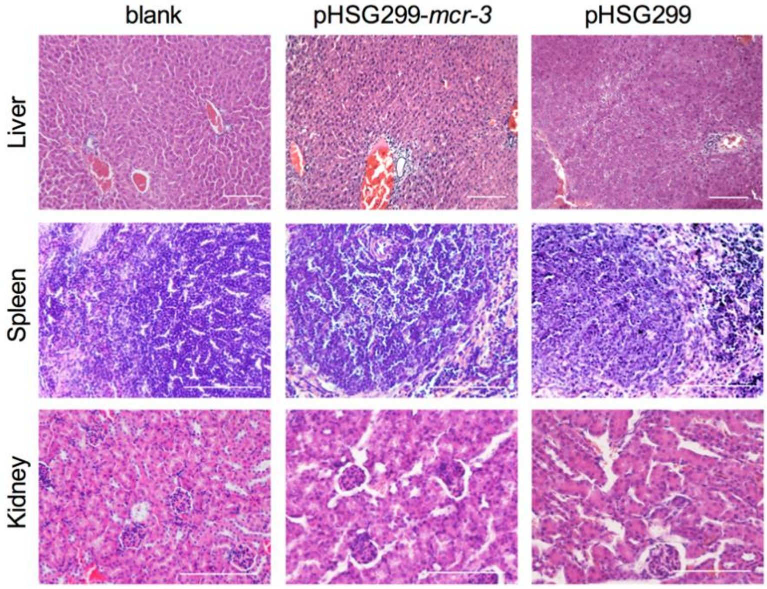

Figure S3 Tissue slices of organs from mice infected with *mcr-3*-positive/negative *A. salmonicida*

Mild congestion and cellular swelling were observed in the liver, along with infiltration of inflammatory cells in experimental groups. No visible pathological signs of bacterial infection were observed in liver sections from control mice. Mild necrosis in some areas of white pulp were observed in the spleen sections from AS1-infected mice. No gross histopathological lesions were observed in the kidney sections of any of the mice.

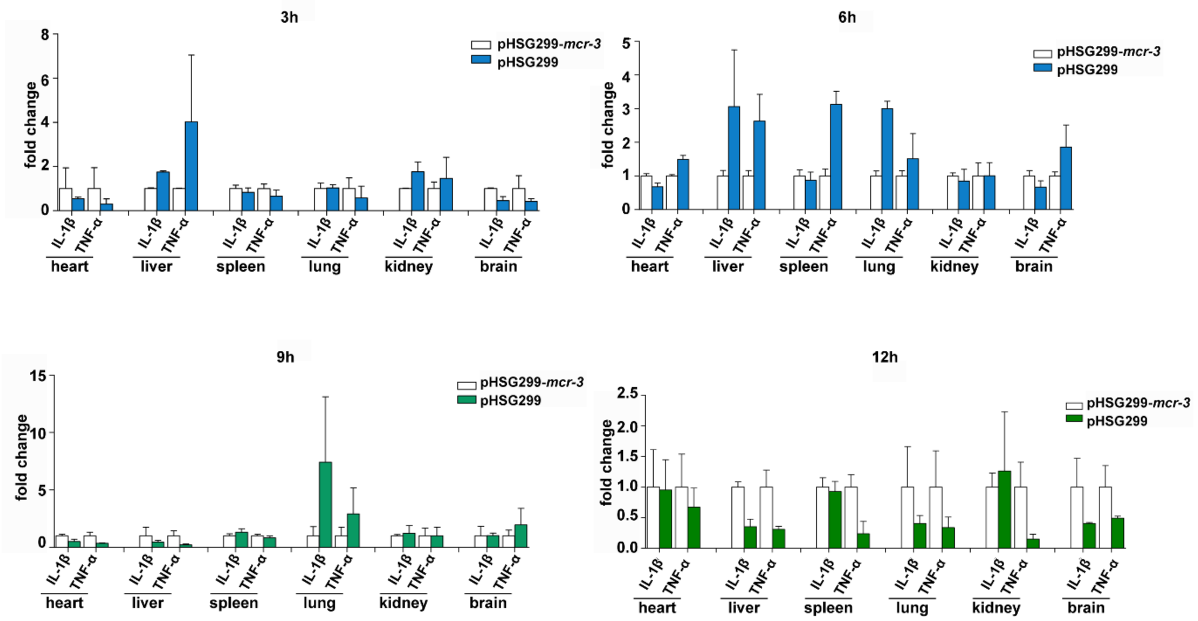

Figure S4 Expression of cytokine genes in organs (heart, liver, spleen, lung, kidney, and brain) from mice infected with *mcr-3*-positive/negative *A. salmonicida* strains at 3, 6, 9, and 12 h post-infection. Results indicate the means ± SEM (n=3).

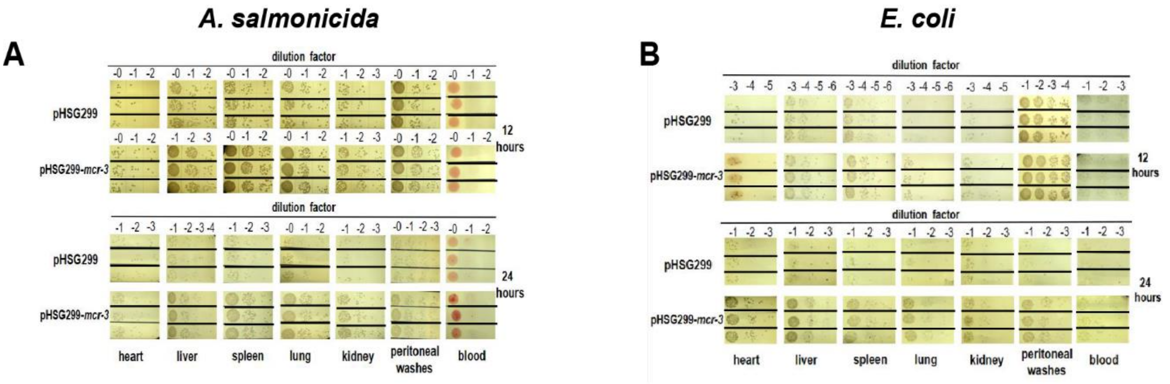

Figure S5 (A, B) Aliquots (10 µL) of blood, peritoneal washes, and tissue homogenate were serially diluted and spotted on LB agar (three mice were tested at 12h and 24 h post-intraperitoneal injection). Images were taken following overnight incubation at 30°C (*A. salmonicida*) or 37°C (*E. coli*).

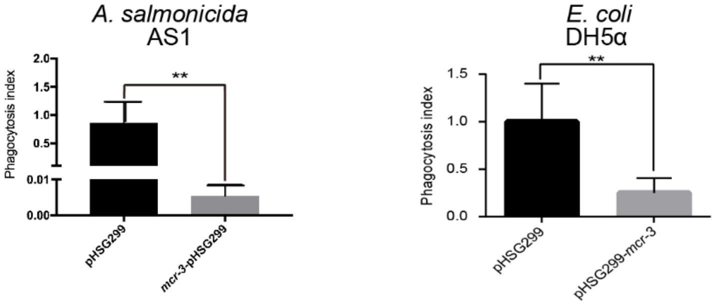

Figure S6 Results were determined by plate counting of intracellular *A. salmonicida* AS1 and *E. coli* DH5α. Results indicate the means ± SEM (n=3).

References

[1] Z. Ling, W. Yin, H. Li, Q. Zhang, X. Wang, Z. Wang, Y. Ke, Y. Wang, J. Shen. *Antimicrob. Agents. Chemother.* **2017**, *61*, e01272.

[2] Y. Shen, C. Xu, Q. Sun, S. Schwarz, Y. Ou, L. Yang, Z. Huang, I. Eichhorn, T. R. Walsh, Y. Wang, R. Zhang, J. Shen. *Antimicrob. Agents. Chemother.* **2018**, *62*, e404.

[3] J. Li, X. Shi, W. Yin, Y. Wang, Z. Shen, S. Ding, S. Wang. *Front. Microbiol.* **2017**, *8*, doi: 10.3389.

[4] W. Gaze, L. Zhang, N. Abdouslam, P. Hawkey, L. Calvo-Bado, J. Royle, H. Brown, S. Davis, P. Kay, A. Boxall, E. Wellington. *ISME* **2011**, *5*, 1253.

[5] S. Ma, C. Sun, A. Hulth, J. Li, L. Nilsson, Y. Zhou, S. Börjesson, Z. Bi, Z. Bi, Q. Sun, Y. Wang. *J. Antimicrob. Chemother.* **2018**, *73*, 1777.

[6] G. Weber, B. Chousterman, S. He, A. Fenn, M. Nairz, A. Anzai, T. Brenner, F. Uhle, Y. Iwamoto, C. Robbins, L. Noiret, S. Maier, T. Zönnchen, N. Rahbari, S. Schölch, A. Ameln, T. Chavakis, J. Weitz, S. Hofer, M. Weigand, M. Nahrendorf, R. Weissleder, F. Swirski. *Science.* **2015**, *347*, 1260.
